# Adjuvant Therapy with Immune Checkpoint Inhibitors after Carbon Ion Radiotherapy for Mucosal Melanoma of the Head and Neck: A Case-Control Study

**DOI:** 10.3390/cancers16152625

**Published:** 2024-07-23

**Authors:** Nobutaka Mizoguchi, Kio Kano, Tatsuya Okuda, Hiroaki Koge, Satoshi Shima, Keisuke Tsuchida, Yosuke Takakusagi, Shohei Kawashiro, Manatsu Yoshida, Yuka Kitani, Kaori Hashimoto, Madoka Furukawa, Katsuyuki Shirai, Tadashi Kamada, Daisaku Yoshida, Hiroyuki Katoh

**Affiliations:** 1 Department of Radiation Oncology, Kanagawa Cancer Center, Yokohama 241-8515, Japan; k-kanou@kcch.jp (K.K.); t-okuda@kcch.jp (T.O.); h-kooge@kcch.jp (H.K.); s-shima@kcch.jp (S.S.); ketsuchi@kcch.jp (K.T.); r5-takakusagi@yokohamasakae.jp (Y.T.); kawashiro.gc60n@kanagawa-pho.jp (S.K.); t-kamada@kcch.jp (T.K.); d.yoshida@kcch.jp (D.Y.); hkatoh@kcch.jp (H.K.); 2 Department of Radiology, Jichi Medical University Saitama Medical Center, Saitama 330-8503, Japan; kshirai@jichi.ac.jp; 3 Department of Head and Neck Surgery, Kanagawa Cancer Center, Yokohama 241-8515, Japan; m-yoshida@kcch.jp (M.Y.); helloidea.ykmr@gmail.com (Y.K.); hckaori@yahoo.co.jp (K.H.); madoka@yokohama.email.ne.jp (M.F.); 4 Department of Radiation Oncology, Jichi Medical University Hospital, Tochigi 329-0498, Japan

**Keywords:** mucosal melanoma, head and neck cancer, carbon ion radiotherapy, immune checkpoint inhibitors, nivolumab, adjuvant therapy, quality of life, cost–benefit analysis

## Abstract

**Simple Summary:**

Mucosal malignant melanoma is a type of head and neck cancer with a high mortality rate. Although favorable local control has been reported when using carbon ion radiotherapy (CIRT) to treat mucosal malignant melanoma of the head and neck, the prognosis remains unsatisfactory because of the tendency for early distant metastasis. In recent years, favorable outcomes have been reported for the use of immune checkpoint inhibitors (ICIs) as adjuvant therapy for cutaneous malignant melanoma, indicating their potential applicability to mucosal malignant melanoma. In addition to achieving favorable local control with CIRT, the control of distant metastasis using ICIs is expected to prolong survival. The findings of this analysis indicate that the use of ICIs as adjuvant therapy can improve prognosis following CIRT, offering a new treatment option.

**Abstract:**

The development of new treatment strategies to improve the prognosis of mucosal malignant melanoma of the head and neck (MMHN) after carbon ion radiotherapy (CIRT) is essential because of the risk of distant metastases. Therefore, our objective was to evaluate the outcomes of immune checkpoint inhibitor (ICI) treatment to justify its inclusion in the regimen after CIRT. Thirty-four patients who received CIRT as an initial treatment were included in the analysis and stratified into three groups: those who did not receive ICIs (Group A), those who received ICIs after recurrence or metastasis (Group B), and those who received ICIs as adjuvant therapy after CIRT (Group C). In total, 62% of the patients (n = 21) received ICIs. The 2-year local control and overall survival (OS) rates for all patients were 90.0% and 66.8%, respectively. The 2-year OS rates for patients in Groups A, B, and C were 50.8%, 66.7%, and 100%, respectively. No significant differences were observed between Groups A and B (*p* = 0.192) and Groups B and C (*p* = 0.112). However, a significant difference was confirmed between Groups A and C (*p* = 0.017). Adjuvant therapy following CIRT for MMHN may be a promising treatment modality that can extend patient survival.

## 1. Introduction

Malignant melanoma is a tumor that arises from melanocytes and is most often diagnosed as skin cancer [1]. It is also found in mucous membranes, ocular choroid, and pia mater [2]. In Europe, the annual incidence rate of malignant melanoma is 1.5 per 1,000,000 individuals, making it an extremely rare tumor. Mucosal malignant melanoma accounts for only 0.7–3.8% of all malignant melanomas [3,4,5,6]. In the head and neck region, it is most frequently detected in the paranasal and nasal sinuses, followed by the oral cavity and pharyngeal mucosa [2,7,8]. Mucosal malignant melanoma has a rapid clinical course, with a 5-year survival rate of 25–46%, even after complete surgical resection, making it one of the head and neck cancers with the highest mortality rate [5,9,10]. In the guidelines developed in the UK and US, surgery is the primary treatment modality, followed by radiotherapy and drug therapy [11,12]. Postoperative irradiation is effective in improving local control; however, it is often reported that it does not contribute to extended survival [13,14,15,16,17,18,19]. The lack of a difference in survival rates following adjuvant therapy emphasizes the need to reassess current treatment approaches for malignant melanoma of the head and neck (MMHN). Moreover, the observation of distant metastasis as the primary cause of treatment failure indicates that novel systemic therapies hold greater promise than postoperative radiotherapy [20,21,22].

In a randomized phase III trial (CheckMate 238 trial) that evaluated the effectiveness of adjuvant therapy in completely resected cases of malignant melanoma (most cases were cutaneous in origin), the use of nivolumab as postoperative adjuvant therapy demonstrated a longer recurrence-free survival and distant metastasis-free survival (DMFS) period than postoperative adjuvant therapy with ipilimumab [23,24]. Although a therapeutic treatment specifically targeting mucosal malignant melanoma has not been administered in randomized controlled trials, the aforementioned CheckMate 238 trial had a small subset of cases where mucosal malignant melanoma comprised approximately 3% of the total cases. This inclusion indicates that adjuvant therapy with nivolumab is efficient in mucosal melanoma treatment. 

Carbon ion radiotherapy (CIRT) has superior dose distribution, higher linear energy transfer, and higher relative biological effectiveness (RBE) compared to photon radiation therapy. According to a multicenter retrospective observational study (J-CROS 1402HN) focusing on MMHN treated with CIRT, the 5-year local control (LC) and overall survival (OS) were 72.3% and 44.6%, respectively [25]. Nevertheless, distant metastases occur frequently, and despite relatively favorable LC, the OS outcomes are not satisfactory. It is necessary to develop novel therapeutic strategies to achieve further improvements. To date, there has been a limited number of reports evaluating the efficacy of adjuvant therapy with immune checkpoint inhibitors (ICIs) in cases of MMHN patients treated with CIRT. In this study, the efficacy, safety, quality of life (QOL), and cost-effectiveness of ICIs were assessed to understand the significance of adjuvant therapy use after CIRT.

## 2. Materials and Methods

### 2.1. Patients and Methods

**Figure 1:**
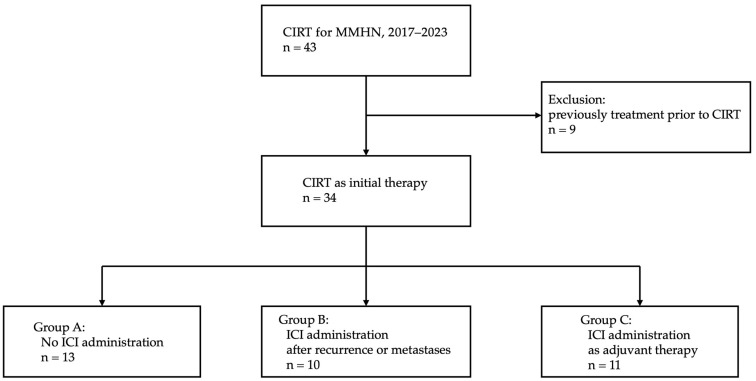
Flowchart for case selection in this study. CIRT, carbon ion radiotherapy; MMHN, mucosal melanoma of the head and neck; ICI, immune checkpoint inhibitor.

This retrospective study analyzed the clinical data of patients with locally advanced MMHN who underwent CIRT at our institution between May 2017 and November 2023. In March 2024, the clinical data were collected. The inclusion criteria for CIRT were as follows: (1) histologically confirmed MMHN, except for choroidal melanoma; (2) N0 or N1 and M0 status; (3) medically inoperable cases or patients who refused surgery; and (4) an Eastern Cooperative Oncology Group (ECOG) performance status of 0–2. This study was approved by the hospital institutional review board (approval number: 2023eki-66). Clinical stages were classified according to the TNM Classification of Malignant Tumors, 8th edition. The staging was determined using computed tomography (CT), magnetic resonance imaging (MRI), ultrasound, positron emission tomography–CT, and physical examination. In this study, cases involving treatment (e.g., surgery or drug therapy) before CIRT were excluded from the analysis (Figure 1). Our treatment strategy for unresectable MMHN is nivolumab administration following CIRT. In cases in which nivolumab administration was not recommended because of factors such as advanced age, poor performance status, and refusal by patients, ICI administration was reconsidered upon recurrence or metastasis. Cases were stratified into those who did not receive ICI therapy (Group A), those involving ICI therapy after recurrence or metastases (Group B), and those incorporating ICI treatment as adjuvant therapy after CIRT (Group C) (Figure 1). 

### 2.2. CIRT

CIRT was administered at a total dose of 64.0 Gy (RBE). This was divided into 16 fractions and performed for 4 weeks. For N1 cases, irradiation was administered simultaneously to both the primary tumor and the metastatic lymph nodes. We did not perform prophylactic radiotherapy of the lymphatic region. Dose calculation and optimization were performed using Monaco version 5.20 (Elekta Solutions AB, Stockholm, Sweden).

### 2.3. ICI Therapy

Nivolumab was used for ICI treatments as adjuvant therapy after CIRT. We administered 240 mg every 2 weeks or 480 mg every 4 weeks. The ICIs used for patients with local recurrence or distant metastases were either a combination of nivolumab and ipilimumab or nivolumab alone. Nivolumab/ipilimumab combination therapy consisted of four courses of 80 mg of nivolumab and 3 mg/kg of ipilimumab every 3 weeks, followed by the administration of nivolumab alone at 240 mg every 2 weeks or 480 mg every 4 weeks.

### 2.4. Evaluation

Treatment efficacy was assessed using the Revised RECIST guideline (version 1.1). Adverse events were evaluated using the Common Terminology Criteria for Adverse Events, version 5.0. Adverse events that occurred within 6 months after CIRT initiation were defined as acute events, whereas those that occurred after 6 months were defined as late events. We assessed adverse events and treatment efficacy on a weekly basis during radiation therapy, on a monthly basis for 1 year following CIRT, every 2–3 months during the second year, and every 3–4 months from the third year onward. The evaluation of treatment efficacy via CT and MRI was performed every 2–3 months.

### 2.5. QOL Analyses

QOL analyses were conducted using EQ-5D-5L (EuroQol-5-Dimensions-5-Level, EuroQol, Rotterdam, The Netherlands) and EQ VAS (EuroQol Visual Analogue Scale) [26,27,28]. The baseline point was defined as that before CIRT initiation, and EQ-5D-5L and EQ VAS scores were evaluated at 1, 3, 6, 9, 12, 15, 18, 21, and 24 months after CIRT initiation.

### 2.6. Cost Parameters

Medical expenses were calculated from the consultation date at the Department of Radiation Oncology, Kanagawa Cancer Center, to 29 February 2024. Quality-adjusted life year (QALY) was calculated by multiplying the QOL value, which was quantified based on the EQ-5D-5L, according to the survival period. The incremental cost-effectiveness ratio (ICER) was evaluated based on the incremental cost and QALYs.

### 2.7. Statistical Analysis

Patient characteristics were compared using the chi-square test. Cumulative OS, LC, DMFS, and progression-free survival (PFS) were estimated using the Kaplan–Meier method. The observation period was defined as beginning with the starting date of the CIRT. All the statistically significant (*p* < 0.05) factors assessed in the univariate analysis were reflected in the Cox proportional hazards model. The comparison of pretreatment and post-CIRT EQ-5D-5L and EQ VAS scores was performed using the Wilcoxon signed-rank test. Statistical tests were performed using SPSS version 26 (IBM, New York, NY, USA).

## 3. Results

From 2017 to 2023, a total of 43 patients with a diagnosis of MMHN received CIRT at our institution. Patients who underwent surgery or drug therapy before CIRT were excluded from the analysis (Figure 1). There were 34 cases evaluated, and the patient characteristics are presented in Table 1. The median follow-up duration for all patients was 17.4 months (range: 3.4–66.0 months), and that for survivors was 26.6 months (range: 3.4–66.0 months).

### 3.1. OS

**Figure 2:**
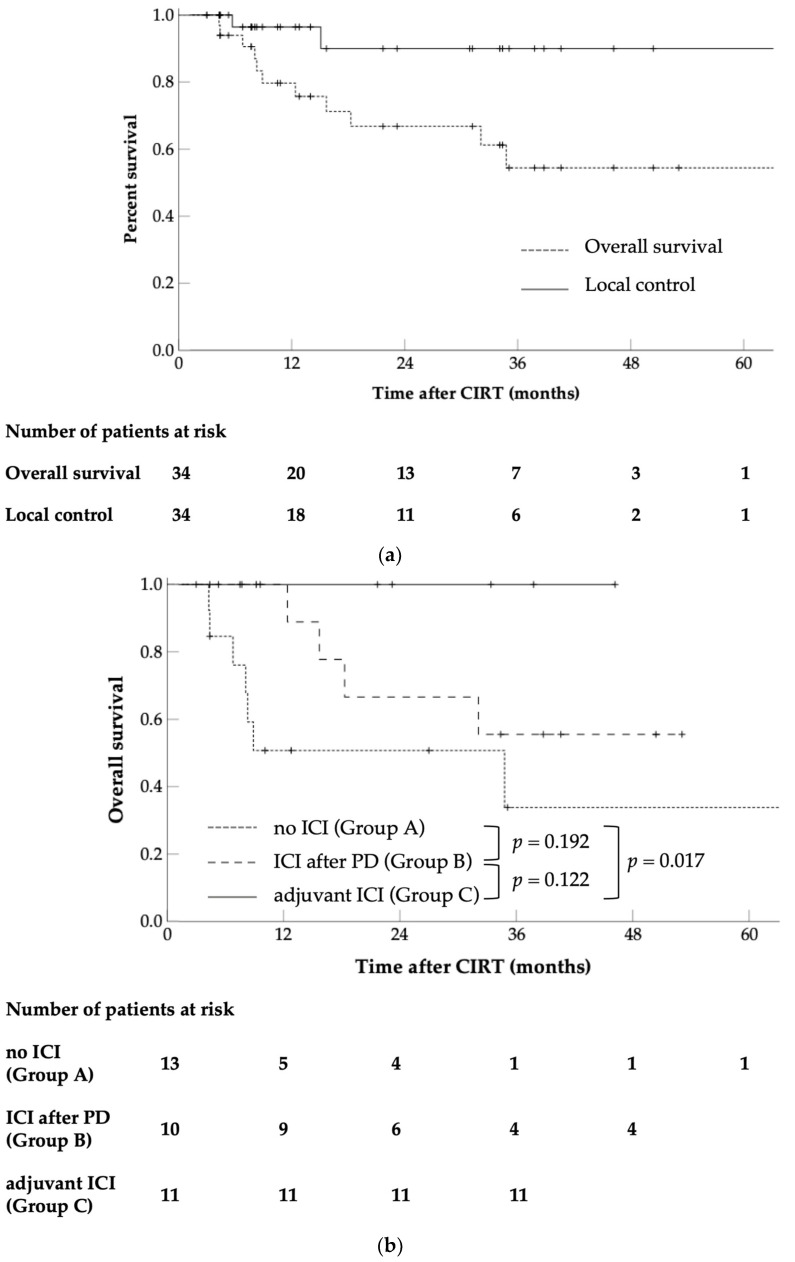
Kaplan–Meier curves of (**a**) overall survival and local control rates for all cases, and (**b**) overall survival by group. CIRT, carbon ion radiotherapy; ICI, immune checkpoint inhibitor; PD, progressive disease.

The OS of all patients is shown in Figure 2a. The OS of patients that were classified according to whether they received ICI and the timing of administration is shown in Figure 2b. A total of ten patients died, of which eight died from cancer and two from conditions unrelated to the disease (one from pneumonia and one from drowning). The OS rates for all patients were 79.7% and 66.8% at 1 and 2 years, respectively (Figure 2a). For those who received ICI therapy, the 1- and 2-year OS rates were 100% and 78.6%, respectively; for those who had not undergone ICI treatment (Group A), the OS rates were 50.8% and 50.8%, respectively. A significant intergroup difference was observed (*p* = 0.020, Supplementary Figure S1). The 1- and 2-year OS rates for patients who received ICI after local recurrence or distant metastases (Group B) were 100% and 66.7%, respectively.

The 1- and 2-year OS values for patients treated with ICIs as adjuvant therapy after CIRT (Group C) were 100%, and those for patients treated with ICIs after progressive disease (Group B) were 100% and 66.7%, respectively. In terms of OS, no significant difference was detected between Groups B and C (*p* = 0.122, Figure 2b) or between Groups A and B (*p* = 0.192). However, a significant difference was found between Groups A and C (*p* = 0.017).

### 3.2. LC

Local recurrence was observed in two patients 5.7 and 15.1 months after CIRT, respectively. The 1- and 2-year LC rates for all patients were 96.4% and 90.0%, respectively (Figure 2a). For patients treated with adjuvant ICIs, the LC rates were both 100%, whereas for those not subjected to ICI treatment, the rates were 95.0% and 85.5%, respectively, reflecting that there was no significant difference (*p* = 0.343, Supplementary Figure S2a).

### 3.3. DMFS and PFS

The 1- and 2-year DMFS rates of patients treated with adjuvant ICIs (Group C) were 100% and 80%, respectively, whereas those not administered with ICIs were 40.6% and 35.5%, respectively, indicating a significant difference (*p* = 0.008, Supplementary Figure S2b). Similarly, the 1- and 2-year PFS rates of patients who underwent adjuvant ICIs (Group C) were 100% and 80%, respectively, compared with 37.7% and 33.0%, respectively, for patients who did not undergo adjuvant ICIs (*p* = 0.004, Supplementary Figure S2c).

### 3.4. Univariate Analysis of OS

Univariate analysis, which encompassed the parameters of age, sex, ECOG PS, tumor location, tumor stage, tumor diameter, PD-L1 status, BRAF mutation, operability, and PTV volume, was performed to identify the risk factors associated with OS (Table 2). Among all patients, age ≥72 years and unresectability were considered risk factors for survival (*p* = 0.004 and 0.033, respectively). For patients who did not undergo ICI therapy, unresectability was a poor prognostic factor (*p* = 0.035).

### 3.5. Acute and Late Adverse Events

The adverse events are summarized in Table 3. Grade 3 mucositis, tumor hemorrhage, and pneumonitis were observed in two (6%) patients, two (6%) patients, and one (6%) patient, respectively, as acute events. Grade 3 late effects were observed in five patients (15%). We classified grade 3 pneumonitis, occurring in acute events, and grade 3 soft tissue infections, occurring in late events, as immune-related adverse events (irAEs) caused by ICI therapy. No grade 4 or higher acute or late adverse events were detected. Adverse events that occurred due to the presence or absence of ICI therapy are presented in Supplementary Table S1.

### 3.6. QOL Analysis

The mean and standard deviation of the QOL values, determined according to the EQ-5D-5L and EQ VAS, are presented in Table 4, with no significant changes in QOL values found.

### 3.7. Cost-Effectiveness Analysis

The QALYs and ICERs assessed in the groups with and without ICI administration are shown in Table 5. The ICER, calculated using the values of incremental medical costs and QALYs, was JPY2,777,404/QALY for patients treated with ICIs. The ICER for patients who were prescribed nivolumab as adjuvant therapy (Group C) was JPY4,534,904/QALY, and for patients treated with ICIs after local recurrence or distant metastases (Group B) it was JPY2,140,557/QALY.

## 4. Discussion

Because MMHN is a very rare disease entity, large-scale clinical trials have not been conducted on it. Therefore, surgical resection is regarded as the standard treatment, as shown in retrospective analyses involving a small number of cases [10,13,14,15,17,29,30]. The lack of difference in survival after adjuvant therapy underscores the need to re-evaluate current treatment approaches to MMHN. Furthermore, the observation that distant metastasis is the main cause of treatment failure indicates that novel systemic therapy is more promising than postoperative radiation therapy. In both British and American guidelines, surgical excision is the primary treatment option; however, for cases with more advanced stage, clinical trials are recommended [11,12].

In recent years, evidence has accumulated in favor of the use of ICIs, particularly for the treatment of cutaneous malignant melanoma. In a randomized phase III trial (CheckMate 238 study) in which either nivolumab or ipilimumab were administered to patients with stage III–IV melanoma who had undergone complete resection, the nivolumab arm had a favorable recurrence-free survival rate and fewer adverse events [23,24]. Because the above clinical trials included mucosal malignant melanoma in a small number of cases, treatment strategies for cutaneous malignant melanoma were often applied to mucosal malignant melanoma. In the past few years, retrospective analyses of some cases treated with ICIs have been conducted for resectable MMHN [20,31,32,33,34]. Patients treated with ICIs after recurrence following surgery or surgery, followed by adjuvant radiotherapy, have been reported to have significantly improved prognosis compared with patients who did not receive ICI [20,33]. Conversely, Jacques et al. examined the significance of adjuvant ICI therapy following surgery and reported no improvement in OS, DMFS, or PFS with adjuvant ICIs [32]. Therefore, the significance of ICI treatment remains controversial, and no definitive conclusion has been reached.

Definitive photon radiotherapy has been attempted for locally advanced or inoperable diseases. However, because MMHN is a radioresistant tumor, adequate therapeutic efficacy has not been achieved [2,35,36,37]. CIRT has high biological effectiveness compared to X-rays and protons, resulting in favorable therapeutic effects against radioresistant tumors [38,39,40]. In a multicenter retrospective observational study J-CROS1402HN involving 260 patients treated with CIRT, approximately 67% of cases were T4, and unresectable cases comprised 66% of the sample; however, the 2-year OS was 69.4%, LC was 83.9%, and PFS was 40.4% [25]. Despite the involvement of advanced-stage cases, the outcomes were comparable to those of surgical cases, indicating that CIRT is a highly effective treatment modality for MMHN. Nonetheless, PFS and OS are not sufficient for analysis because of the early occurrence of distant metastasis; therefore, it is necessary to develop a relevant treatment strategy.

In this study, the significance of ICIs was examined, which implied survival rate improvement while maintaining favorable LC with CIRT. The 2-year OS was 78.6% for patients treated with ICIs and 50.8% for those who did not undergo ICI therapy, where the results were significantly more promising in the ICI group (*p* = 0.022), indicating that ICI treatment is effective in prolonging survival. Of the 21 patients who received ICI treatment, 11 received ICIs as adjuvant therapy after CIRT and 10 were treated after recurrence or metastasis was documented. All patients who underwent adjuvant therapy survived and did not develop local recurrence. Distant metastasis was noted in only one case. The 2-year OS rates of patients who received and did not receive adjuvant therapy with ICIs were 100% and 50.8%, respectively, indicating a significant difference (Figure 2b, *p* = 0.017). However, no significant difference was observed in OS between patients who received ICIs after recurrence or metastasis and those who did not (Figure 2b, *p* = 0.192). Although no significant difference in LC was observed between patients who received and did not receive adjuvant ICI therapy (Supplementary Figure S2a, *p* = 0.343), these groups differed significantly in terms of DMFS (Supplementary Figure S2b, *p* = 0.008), indicating that adjuvant ICIs are effective in controlling distant metastases. These findings support the conclusion that the administration of adjuvant ICI therapy before recurrence or metastasis is important for prolonging patient survival. Among all patients, age ≥ 72 years and unresectability were considered risk factors for survival (*p* = 0.004 and 0.033, respectively). In patients who did not receive ICIs, unresectability remained a risk factor, and patients aged >72 years tended toward poor prognosis (*p* = 0.035 and 0.075, respectively). However, in patients receiving ICIs, neither age nor resectability were risk factors, indicating the possibility of performing ICI administration to determine prognosis.

CIRT is associated with a risk of serious late complications. Koto et al. reported grade 3 or higher late adverse events in 33 (13%) out of 260 patients treated with CIRT for MMHN [25]. Furthermore, a multicenter study on adenoid cystic carcinoma of the head and neck treated with CIRT demonstrated grade 3 or higher late adverse events in 15% of patients [41]. Owing to the high biological effectiveness of CIRT, the incidence of late effects, such as jaw osteonecrosis, oral cavity fistula, and brain necrosis, is increased in patients with severe jawbone, palate, and skull base invasion. In the present analysis of 34 cases, grade 3 late adverse events were detected in five cases (15%) (Table 3). Soft tissue infection (3%) was considered an irAE, and the remaining four cases (12%) were defined as CIRT-related adverse events. No unexpected adverse events were observed, indicating a favorable safety profile for ICI administration.

Cancer treatment remains controversial for older adults. Patients with various comorbidities and reduced physical function and organ reserve, mostly comprising this group, are at increased risk for invasive treatment, such as surgical resection; thus, the development of a strategy that preserves the patient’s QOL is warranted [42]. QOL values from before CIRT initiation were used as the baseline, and changes were measured periodically for up to 2 years thereafter. Significant differences in QOL scores were not detected at each time point (Table 4). Sprave et al. showed a similar finding in their analysis of 336 patients treated with photon therapy for head and neck cancer, with no significant decreases in EQ-5D-5L or VAS scores at the end of radiotherapy [43]. The lack of an apparent decline in QOL values indicates that high-precision radiation therapy, such as CIRT and intensity-modulated radiation therapy, has become a well-established technology and that strict control has been achieved of radiation therapy-related adverse effects. Our results indicate that CIRT is feasible and does not reduce QOL in patients with inoperable tumors aggravated by comorbidities and impaired organ function.

Using cases without ICIs as the control group (Group A), the incremental cost and QALYs were evaluated (Table 5). Patients receiving ICIs as adjuvant therapy experienced an increase in QALYs; however, the ICER also increased (JPY4,534,904/QALY [€26,759/QALY, $28,639/QALY]). There are no clear reports examining the cost-effectiveness of ICId as adjuvant therapy after definitive radiotherapy for mucosal malignant melanoma. For patients treated with nivolumab as adjuvant therapy after surgery in the CheckMate 238 and KEYNOTE-054 trials, the calculated ICERs were €21,153/QALY and $98,112/QALY (€89,488/QALY) [23,44,45,46]. ICER does not have a clearly defined criterion or threshold; however, it is considered “cost effective” if it is up to JPY 5 million/JPY 10 million in Japan, £20,000–£30,000 in the UK [47], and $104,000 in the US [48]. The World Health Organization recommends a cost-effectiveness threshold of 1–3 times the gross domestic product (GDP) per capita and this is often cited when cost-effectiveness thresholds are discussed [49]. The GDP per capita in Japan is $34,114 (JPY5,393,252, and €31,874) [50]. The ICER for patients undergoing nivolumab adjuvant therapy is 0.8 times the GDP per capita, indicating cost-effectiveness. Some countries, such as the UK, the Netherlands, and Sweden, have established different standards for anticancer drugs and those for intractable diseases, and in Japan, thresholds should be reviewed as appropriate according to changes in social and economic conditions.

Our findings indicate that the use of adjuvant ICI therapy after CIRT for MMHN can be a promising treatment option that extends the OS of patients. However, this study has several limitations. First, the study was designed as a single-center retrospective analysis. Second, the number of enrolled patients was small. Because multivariate analysis was not performed owing to the insufficient number of cases, the existence of OS-related confounding factors could not be ruled out. Third, the administration and timing of ICIs were not randomized. Because MMHN is a rare disease, it is difficult to conduct large-scale prospective clinical trials. Thus, it is necessary to evaluate the role of CIRT followed by adjuvant therapy with ICIs in multicenter retrospective studies, meta-analyses using the results from the multiple studies conducted to date, and cross-sectional analyses using a multicenter database. Currently, we are performing a prospective observational study on adjuvant therapy with nivolumab after CIRT for MMHN (UMIN-CTR Clinical Trial: UMIN000042226) [51].

## 5. Conclusions

Even when achieving good LC of MMHN, overall survival improvement is hampered by the high incidence of subsequent distant metastases. Therefore, new treatment strategies that can prevent distant metastases are needed to improve prognosis. As adjuvant therapy after CIRT for MMHN, nivolumab is a promising new treatment strategy for suppressing distant metastases and prolonging survival in addition to the favorable LC achieved by CIRT.

## Figures and Tables

**Table 1:** Patient characteristics.

		All Cases	Group A *	Group B *	Group C *	*p* Value
		34	13	10	11	
**Age (years)**	**Median (range)**	72 (49–94)	82 (54–94)	71 (55–79)	68 (49–83)	0.009
**Sex**	**Male**	11	4	4	3	0.814
	**Female**	23	9	6	8	
**ECOG PS**	**0**	11	7	3	1	0.164
	**1**	22	6	6	10	
	**2**	1	0	1	0	
**Tumor location**	**Nasal cavity**	24	10	7	7	0.614
	**Paranasal**	8	2	3	3	
	**Oral cavity**	1	1	0	0	
	**Pharynx**	1	0	0	1	
**Tumor stage (UICC 8th)**	**T3**	10	4	2	4	0.707
	**T4**	24	9	8	7	
**Nodal stage (UICC 8th)**	**N0**	32	12	10	10	0.636
	**N1**	2	1	0	1	
**Tumor diameter (mm)**	**Median (range)**	52 (12–75)	54 (12–72)	61 (31–67)	50 (35–75)	0.139
**PD-L1 expression status**	**≥5%**	9	2	3	4	0.165
	**<5%**	7	1	4	2	
	**Unknown**	18	10	3	5	
**BRAF mutation status**	**Wild-type**	15	3	5	7	0.134
	**Mutation**	1	0	1	0	
	**Unknown**	18	10	4	4	
**Operability**	**Yes**	12	3	2	7	0.057
	**No**	22	10	8	4	
**PTV volume (mL)**	**Median (range)**	156.0 (87.5–212.7)	151.2 (87.5–197.4)	153.8 (111.2–189.9)	156.2 (121.0–212.7)	0.920

* Group A, cases without ICI administration; Group B, cases administered with ICIs after recurrence or metastasis; Group C, cases administered with ICIs as adjuvant therapy.

**Table 2:** Univariate analysis of overall survival.

			All Cases		Group A *		Group B *		Group C *	
		n	2-yr OS (%)	*p* Value	2-yr OS (%)	*p* Value	2-yr OS (%)	*p* Value	2-yr OS (%)	*p* Value
**Age**	**<72**	16	91.7	ref.	100	ref.	83.3	ref.	100	ref.
	**≥72**	18	42.0	0.004	40.9	0.078	33.3	0.266	100	-
**Sex**	**Male**	11	79.5	ref.	50.0	ref.	100	ref.	100	ref.
	**Female**	23	60.3	0.316	50.8	0.866	50.0	0.093	100	-
**ECOG PS**	**0**	23	70.0	ref.	53.6	ref.	60.0	ref.	100	ref.
	**1/2**	11	62.3	0.202	50.0	0.714	75.0	0.899	100	-
**Tumor location**	**Nasal cavity**	24	66.7	ref.	67.5	ref.	57.1	ref.	100	ref.
	**others**	10	64.3	0.908	0.0	0.090	100	0.219	100	-
**Tumor stage**	**T3**	10	78.8	ref.	75.0	ref.	50.0	ref.	100	ref.
	**T4a/T4b**	24	59.5	0.115	38.1	0.164	71.4	0.928	100	-
**Tumor diameter**	**<52 mm**	17	76.4	ref.	62.5	ref.	50.0	ref.	100	ref.
	**≥52 mm**	17	60.2	0.227	42.9	0.400	71.4	0.642	100	-
**PD-L1 status**	**≥5%**	9	65.6	ref.	50.0	ref.	50.0	ref.	100	ref.
	**<5%**	7	83.3	0.177	-	0.676	75.0	0.351	100	-
	**unknown**	18	51.4		45.0		33.3		100	
**BRAF mutation**	**Yes**	1	100	ref.	-	ref.	-	ref.	-	ref.
	**No**	15	92.9	0.086	66.7	0.728	100	0.156	100	-
	**unknown**	18	60.7		45.0		25.0		100	
**Operability**	**Resectable**	12	87.5	ref.	100	ref.	50.0	ref.	100	ref.
	**Unresectable**	12	56.4	0.033	34.3	0.035	71.4	0.928	100	-
**PTV volume**	**<156 mL**	17	46.3	ref.	28.6	ref.	25.0	ref.	100	ref.
	**≥156 mL**	17	92.3	0.095	80.0	0.149	100	0.057	100	-

* Group A, cases without ICI administration; Group B, cases administered with ICIs after recurrence or metastasis; Group C, cases administered with ICIs as adjuvant therapy.

**Table 3:** Acute and late adverse events.

	Acute Adverse Event				Late Adverse Event			
	Any Grade	Grades 1–2	Grade 3	Grade 4+	Any Grade	Grades 1–2	Grade 3	Grade 4+
**Mucositis**	33 (97%)	31 (91%)	2 (6%)	0	2 (6%)	2 (6%) #	0	0
**Dermatitis**	27 (79%)	27 (79%)	0	0	1 (3%)	1 (3%)	0	0
**Dry mouth**	8 (24%)	8 (24%)	0	0	2 (6%)	2 (6%)	0	0
**Dysgeusia**	6 (18%)	6 (18%)	0	0	2 (6%)	2 (6%)	0	0
**Tumor hemorrhage**	2 (6%)	0	2 (6%)	0	1 (3%)	0	1 (3%)	0
**Pneumonitis**	1 (3%)	0	1 (3%) *	0	0	0	0	0
**Optic nerve disorder**	0	0	0	0	3 (9%)	3 (9%)	0	0
**Uveitis**	0	0	0	0	2 (6%)	2 (6%) *	0	0
**Cataract**	0	0	0	0	1 (3%)	0	1 (3%)	0
**Keratitis**	0	0	0	0	1 (3%)	1 (3%)	0	0
**Photophobia**	0	0	0	0	1 (3%)	1 (3%)	0	0
**Watering eyes**	0	0	0	0	1 (3%)	1 (3%)	0	0
**Trismus**	0	0	0	0	2 (6%)	2 (6%)	0	0
**Trigeminal nerve disorder**	0	0	0	0	1 (3%)	0	1 (3%)	0
**Tinnitus**	0	0	0	0	2 (6%)	2 (6%)	0	0
**Otitis media**	0	0	0	0	1 (3%)	1 (3%)	0	0
**Hearing impaired**	0	0	0	0	1 (3%)	0	1 (3%)	0
**Oral cavity fistula**	0	0	0	0	1 (3%)	1 (3%)	0	0
**Hypothyroidism**	0	0	0	0	1 (3%)	1 (3%) *	0	0
**Adrenal insufficiency**	0	0	0	0	1 (3%)	1 (3%) *	0	0
**Soft tissue infection**	0	0	0	0	1 (3%)	0	1 (3%) *	0
**Eczema**	0	0	0	0	1 (3%)	1 (3%) *	0	0
**Arthralgia**	0	0	0	0	1 (3%)	1 (3%) *	0	0
**Fever**	0	0	0	0	1 (3%)	1 (3%) *	0	0

* irAEs, immune-related adverse events; # one case was an irAE.

**Table 4:** EuroQol-5-Dimensions-5-Level (EQ-5D-5L) and EuroQol Visual Analogue Scale (EQ VAS) scores at baseline and after CIRT.

		EQ-5D-5L			EQ VAS	
	Mean	SD	*p* Value	Mean	SD	*p* Value
**Baseline**	0.867	0.101	ref.	70.0	16.425	ref.
**1 M**	0.895	0.124	0.794	75.0	16.251	0.242
**3 M**	0.881	0.175	0.537	72.5	19.260	0.536
**6 M**	0.889	0.163	0.972	70.0	14.573	0.081
**9 M**	0.894	0.172	0.208	72.5	14.577	0.326
**12 M**	0.867	0.176	0.310	80.0	19.656	0.779
**15 M**	0.895	0.132	0.753	70.0	10.670	0.609
**18 M**	0.895	0.105	0.825	80.0	16.073	0.786
**21 M**	0.895	0.077	0.715	80.0	14.142	0.578
**24 M**	1.000	0.106	0.415	80.0	12.392	0.680

SD, standard deviation; CIRT, carbon ion radiotherapy; baseline, before CIRT; 1 M, 1 month; 3 M, 3 months; 6 M, 6 months; 9 M, 9 months; 12 M, 12 months; 15 M, 15 months; 18 M, 18 months; 21 M, 21 months; and 24 M, 24 months after CIRT.

**Table 5:** Incremental cost-effectiveness ratio with and without ICIs.

Treatment	Total Costs (JPY)	Incremental Cost (JPY)	QALYs	Incremental QALYs	ICER (JPY/QALY)
**No ICI (Group A)**	2,732,870	ref.	0.642	reference	ref.
**ICI administration**	7,293,875	4,589,010	2.295	1.652	2,777,404
**ICI after PD (Group B)**	6,687,248	3,954,378	2.490	1.847	2,140,557
**Adjuvant ICI (Group C)**	7,600,205	4,867,335	1.716	1.073	4,534,904

ICI, immune checkpoint inhibitor; QALY, quality-adjusted life year; ICER, incremental cost-effectiveness ratio; PD, progressive disease.

## Data Availability

The data presented in this study are available on request from the corresponding author.

## Supplementary Materials

The following supporting information can be downloaded at https://www.mdpi.com/article/10.3390/cancers16152625/s1: Figure S1: Kaplan–Meier curves of overall survival rates with and without ICIs; Figure S2: Kaplan–Meier curves of (a) local control rate, (b) distant metastasis-free survival rate, and (c) progression-free survival rate, with and without adjuvant ICIs; Table S1: Acute and late adverse events with and without ICI administration.

## References

[1] Yde S.S., Sjoegren P., Heje M., Stolle L.B. (2018). Mucosal melanoma: A literature review. Curr. Oncol. Rep..

[2] Ascierto P.A., Accorona R., Botti G., Farina D., Fossati P., Gatta G., Gogas H., Lombardi D., Maroldi R., Nicolai P. (2017). mucosal melanoma of the head and neck. Crit. Rev. Oncol. Hematol..

[3] Chang A.E., Karnell L.H., Menck H.R. (1998). The national cancer data base report on cutaneous and noncutaneous melanoma: A summary of 84,836 cases from the past decade. Cancer.

[4] Lourenco S.V., Fernandes J.D., Hsieh R., Coutinho-Camillo C.M., Bologna S., Sangueza M., Nico M.M. (2014). Head and neck mucosal melanoma: A review. Am. J. Dermatopathol..

[5] Lazarev S., Gupta V., Hu K., Harrison L.B., Bakst R. (2014). Mucosal melanoma of the head and neck: A systematic review of the literature. Int. J. Radiat. Oncol. Biol. Phys..

[6] Gatta G., Capocaccia R., Botta L., Mallone S., De Angelis R., Ardanaz E., Comber H., Dimitrova N., Leinonen M.K., Siesling S. (2017). Burden and centralised treatment in Europe of rare tumours: Results of RARECAREnet-a population-based study. Lancet Oncol..

[7] Marcus D.M., Marcus R.P., Prabhu R.S., Owonikoko T.K., Lawson D.H., Switchenko J., Beitler J.J. (2012). Rising incidence of mucosal melanoma of the head and neck in the United States. J. Skin Cancer.

[8] Jethanamest D., Vila P.M., Sikora A.G., Morris L.G. (2011). Predictors of survival in mucosal melanoma of the head and neck. Ann. Surg. Oncol..

[9] Lopez F., Rodrigo J.P., Cardesa A., Triantafyllou A., Devaney K.O., Mendenhall W.M., Haigentz M., Strojan P., Pellitteri P.K., Bradford C.R. (2016). Update on primary head and neck mucosal melanoma. Head Neck.

[10] Shah J.P., Levyn H., Valero C., Adilbay D., Eagan A., Zheng J., Gonen M., Cohen M., Patel S., Ganly I. (2024). Skull base surgery for malignant tumors: The 2nd international collaborative study (1995–2015). Head Neck.

[11] Nenclares P., Ap Dafydd D., Bagwan I., Begg D., Kerawala C., King E., Lingley K., Paleri V., Paterson G., Payne M. (2020). Head and neck mucosal melanoma: The United Kingdom national guidelines. Eur. J. Cancer.

[12] Guidelines N. Head and Neck Cancers Version 3.2024. https://www.nccn.org.

[13] Owens J.M., Roberts D.B., Myers J.N. (2003). The role of postoperative adjuvant radiation therapy in the treatment of mucosal melanomas of the head and neck region. Arch. Otolaryngol. Head Neck Surg..

[14] Temam S., Mamelle G., Marandas P., Wibault P., Avril M.F., Janot F., Julieron M., Schwaab G., Luboinski B. (2005). Postoperative radiotherapy for primary mucosal melanoma of the head and neck. Cancer.

[15] Meleti M., Leemans C.R., De Bree R., Vescovi P., Sesenna E., Van Der Waal I. (2008). Head and neck mucosal melanoma: Experience with 42 patients, with emphasis on the role of postoperative radiotherapy. Head Neck.

[16] Moreno M.A., Roberts D.B., Kupferman M.E., Demonte F., El-Naggar A.K., Williams M., Rosenthal D.S., Hanna E.Y. (2010). Mucosal melanoma of the nose and paranasal sinuses, a contemporary experience from the M. D. Anderson cancer center. Cancer.

[17] Benlyazid A., Thariat J., Temam S., Malard O., Florescu C., Choussy O., Makeieff M., Poissonnet G., Penel N., Righini C. (2010). Postoperative radiotherapy in head and neck mucosal melanoma: A GETTEC study. Arch. Otolaryngol. Head Neck Surg..

[18] Sun S., Huang X., Gao L., Zhang Y., Luo J., Zhang S., Wang K., Qu Y., Wu R., Liu Q. (2017). Long-term treatment outcomes and prognosis of mucosal melanoma of the head and neck: 161 cases from a single institution. Oral Oncol..

[19] Gal T.J., Silver N., Huang B. (2011). Demographics and treatment trends in sinonasal mucosal melanoma. Laryngoscope.

[20] Sahovaler A., Ziai H., Cardemil F., Huang S.H., Su J., Goldstein D.P., Gilbert R., Hosni A., Hope A., Waldron J. (2021). Importance of margins, radiotherapy, and systemic therapy in mucosal melanoma of the head and neck. Laryngoscope.

[21] Torabi S.J., Benchetrit L., Spock T., Cheraghlou S., Judson B.L. (2019). Clinically node-negative head and neck mucosal melanoma: An analysis of current treatment guidelines & outcomes. Oral. Oncol..

[22] Amit M., Tam S., Abdelmeguid A.S., Kupferman M.E., Su S.Y., Raza S.M., DeMonte F., Hanna E.Y. (2018). Patterns of treatment failure in patients with sinonasal mucosal melanoma. Ann. Surg. Oncol..

[23] Weber J., Mandala M., Del Vecchio M., Gogas H.J., Arance A.M., Cowey C.L., Dalle S., Schenker M., Chiarion-Sileni V., Marquez-Rodas I. (2017). Adjuvant nivolumab versus ipilimumab in resected stage III or IV melanoma. N. Engl. J. Med..

[24] Larkin J., Del Vecchio M., Mandala M., Gogas H., Fernandez A.M., Dalle S., Cowey C.L., Schenker M., Grob J.J., Chiarion-Sileni V. (2023). Adjuvant nivolumab versus ipilimumab in resected stage III/IV melanoma: 5-year efficacy and biomarker results from CheckMate 238. Clin. Cancer Res..

[25] Koto M., Demizu Y., Saitoh J.I., Suefuji H., Tsuji H., Okimoto T., Ohno T., Shioyama Y., Takagi R., Nemoto K. (2017). Multicenter study of carbon-ion radiation therapy for mucosal melanoma of the head and neck: Subanalysis of the Japan Carbon-Ion Radiation Oncology Study Group (J-CROS) Study (1402 HN). Int. J. Radiat. Oncol. Biol. Phys..

[26] Shiroiwa T., Ikeda S., Noto S., Igarashi A., Fukuda T., Saito S., Shimozuma K. (2016). Comparison of value set based on DCE and/or TTO data: Scoring for EQ-5D-5L health states in Japan. Value Health.

[27] van Hout B., Janssen M.F., Feng Y.S., Kohlmann T., Busschbach J., Golicki D., Lloyd A., Scalone L., Kind P., Pickard A.S. (2012). Interim scoring for the EQ-5D-5L: Mapping the EQ-5D-5L to EQ-5D-3L value sets. Value Health.

[28] Herdman M., Gudex C., Lloyd A., Janssen M., Kind P., Parkin D., Bonsel G., Badia X. (2011). Development and preliminary testing of the new five-level version of EQ-5D (EQ-5D-5L). Qual. Life. Res..

[29] Krengli M., Jereczek-Fossa B.A., Kaanders J.H., Masini L., Beldi D., Orecchia R. (2008). What is the role of radiotherapy in the treatment of mucosal melanoma of the head and neck?. Crit. Rev. Oncol. Hematol..

[30] Lee S.P., Shimizu K.T., Tran L.M., Juillard G., Calcaterra T.C. (1994). Mucosal melanoma of the head and neck: The impact of local control on survival. Laryngoscope.

[31] Smart A.C., Giobbie-Hurder A., Desai V., Xing J.L., Lukens J.N., Taunk N.K., Sullivan R.J., Mooradian M.J., Hsu C.C., Buchbinder E.I. (2024). Multicenter evaluation of radiation and immune checkpoint inhibitor therapy in mucosal melanoma and review of recent literature. Adv. Radiat. Oncol..

[32] Jacques S.K., McKeown J., Grover P., Johnson D.B., Zaremba A., Dimitriou F., Weiser R., Farid M., Namikawa K., Sullivan R.J. (2024). Outcomes of patients with resected stage III/IV acral or mucosal melanoma, treated with adjuvant anti-PD-1 based therapy. Eur. J. Cancer.

[33] Lechner M., Takahashi Y., Turri-Zanoni M., Ferrari M., Liu J., Counsell N., Mattavelli D., Rampinelli V., Vermi W., Lombardi D. (2023). International multicenter study of clinical outcomes of sinonasal melanoma shows survival benefit for patients treated with immune checkpoint inhibitors and potential improvements to the current TNM staging system. J. Neurol. Surg. B Skull Base.

[34] Teterycz P., Czarnecka A.M., Indini A., Spalek M.J., Labianca A., Rogala P., Cybulska-Stopa B., Quaglino P., Ricardi U., Badellino S. (2020). Multimodal treatment of advanced mucosal melanoma in the era of modern immunotherapy. Cancers.

[35] Nenclares P., Harrington K.J. (2022). Management of head and neck mucosal melanoma. Oral Maxillofac. Surg. Clin. N. Am..

[36] Rofstad E.K., Wahl A., Tveit K.M., Monge O.R., Brustad T. (1985). Survival curves after X-ray and heat treatments for melanoma cells derived directly from surgical specimens of tumours in man. Radiother. Oncol..

[37] Barranco S.C., Romsdahl M.M., Humphrey R.M. (1971). The radiation response of human malignant melanoma cells grown in vitro. Cancer Res..

[38] Kamada T., Tsujii H., Tsuji H., Yanagi T., Mizoe J.E., Miyamoto T., Kato H., Yamada S., Morita S., Yoshikawa K. (2002). Efficacy and safety of carbon ion radiotherapy in bone and soft tissue sarcomas. J. Clin. Oncol..

[39] Shinoto M., Yamada S., Terashima K., Yasuda S., Shioyama Y., Honda H., Kamada T., Tsujii H., Saisho H., Working Group for Pancreas C. (2016). Carbon ion radiation therapy with concurrent gemcitabine for patients with locally advanced pancreatic cancer. Int. J. Radiat. Oncol. Biol. Phys..

[40] Yamada S., Kamada T., Ebner D.K., Shinoto M., Terashima K., Isozaki Y., Yasuda S., Makishima H., Tsuji H., Tsujii H. (2016). Carbon-ion radiation therapy for pelvic recurrence of rectal cancer. Int. J. Radiat. Oncol. Biol. Phys..

[41] Sulaiman N.S., Demizu Y., Koto M., Saitoh J.I., Suefuji H., Tsuji H., Ohno T., Shioyama Y., Okimoto T., Daimon T. (2018). Multicenter study of carbon-ion radiation therapy for adenoid cystic carcinoma of the head and neck: Subanalysis of the Japan Carbon-Ion Radiation Oncology Study Group (J-CROS) Study (1402 HN). Int. J. Radiat. Oncol. Biol. Phys..

[42] Dickstein D.R., Lehrer E.J., Hsieh K., Hotca A., Jones B.M., Powers A., Sharma S., Liu J., Gupta V., Mell L. (2022). Management of older adults with locally advanced head and neck cancer. Cancers.

[43] Sprave T., Gkika E., Verma V., Grosu A.L., Stoian R. (2022). Patient reported outcomes based on EQ-5D-5L questionnaires in head and neck cancer patients: A real-world study. BMC Cancer.

[44] Eggermont A.M.M., Blank C.U., Mandala M., Long G.V., Atkinson V., Dalle S., Haydon A., Lichinitser M., Khattak A., Carlino M.S. (2018). Adjuvant pembrolizumab versus placebo in resected stage III melanoma. N. Engl. J. Med..

[45] Mulder E., Smit L., Grunhagen D.J., Verhoef C., Sleijfer S., van der Veldt A.A.M., Uyl-de Groot C.A. (2021). Cost-effectiveness of adjuvant systemic therapies for patients with high-risk melanoma in Europe: A model-based economic evaluation. ESMO Open.

[46] Mojtahed S.A., Boyer N.R., Rao S.A., Gajewski T.F., Tseng J., Turaga K.K. (2021). Cost-effectiveness analysis of adjuvant therapy for BRAF-mutant resected stage III melanoma in Medicare patients. Ann. Surg. Oncol..

[47] National Institute for Health and Care Exellence-NICE Health Technology Evaluations: The Manual. https://www.nice.org.uk/process/pmg36/chapter/introduction-to-health-technology-evaluation.

[48] Vanness D.J., Lomas J., Ahn H. (2021). A health opportunity cost threshold for cost-effectiveness analysis in the United States. Ann. Intern. Med..

[49] World Health Organization (2002). World Health Report: 2002, Reducing Risks, Promoting Healthy Life. https://www.who.int/publications/i/item/9241562072.

[50] Department of Economic and Social Affairs, United Nations National Accounts-Analysis of Main Aggregates (AMA). https://unstats.un.org/UNSDWebsite/.

[51] UMIN-CTR Clinical Trial. https://center6.umin.ac.jp/cgi-open-bin/ctr_e/ctr_view.cgi?recptno=R000048210.

