# Supplementary material for: Adjuvant Therapy with Immune Checkpoint Inhibitors after Carbon Ion Radiotherapy for Mucosal Melanoma of the Head and Neck: A Case-Control Study
Source: Cancers (Basel). 2024 Jul 23;16(15):2625. doi: 10.3390/cancers16152625 (PMC11311030; doi:10.3390/cancers16152625)
Supplement: Supplementary file 1 [file cancers-16-02625-s001.zip › Figures S1 and S2.pdf]

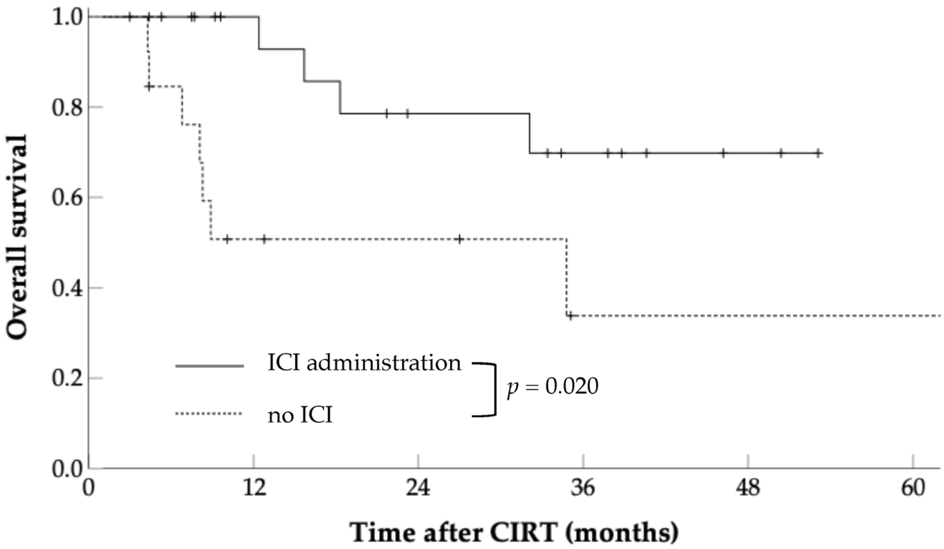


**Number of patients at risk**

| | | | | | | |
|---|---|---|---|---|---|---|
| **ICI administration (Group B + Group C)** | **21** | **14** | **9** | **7** | **2** | |
| **no ICI (Group A)** | **13** | **5** | **4** | **1** | **1** | **1** |

**Figure S1.** Kaplan–Meier curves of overall survival rates with and without ICIs.

(a)

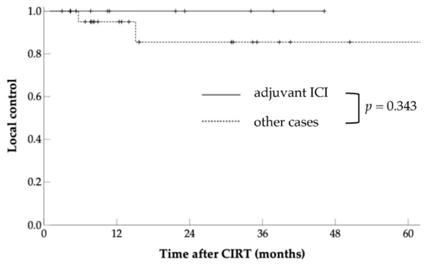


**Number of patients at risk**

| | | | | | | |
|---|---|---|---|---|---|---|
| **adjuvant ICI** | **11** | **5** | **3** | **3** | | |
| **other cases** | **23** | **13** | **8** | **4** | **2** | **1** |

(b)

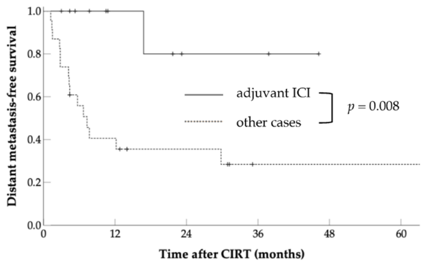


**Number of patients at risk**

| | | | | | | |
|---|---|---|---|---|---|---|
| **adjuvant ICI** | **11** | **5** | **2** | **2** | | |
| **other cases** | **23** | **8** | **5** | **1** | **1** | **1** |

(c)

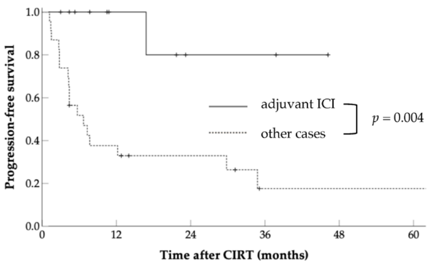


**Number of patients at risk**

| | | | | | | |
|---|---|---|---|---|---|---|
| **adjuvant ICI** | **11** | **5** | **2** | **2** | | |
| **other cases** | **23** | **8** | **5** | **1** | **1** | **1** |

**Figure S2.** Kaplan–Meier curves of (**a**) local control rate, (**b**) distant metastasis-free survival rate, and (**c**) progression-free survival rate, with and without adjuvant ICIs.
