# Supplementary material for: Adjuvant Therapy with Immune Checkpoint Inhibitors after Carbon Ion Radiotherapy for Mucosal Melanoma of the Head and Neck: A Case-Control Study
Source: Cancers (Basel). 2024 Jul 23;16(15):2625. doi: 10.3390/cancers16152625 (PMC11311030; doi:10.3390/cancers16152625)
Supplement: Supplementary file 1 [file cancers-16-02625-s001.zip › Table S1.pdf]

Table S1. Acute and late adverse events with and without ICI administration.

	All cases (n = 34)				ICI (−), Group A (n = 13)				ICI (+), Group B + C (n = 21)			
**Acute adverse event**	**Any Gr**	**Gr 1–2**	**Gr 3**	**Gr 4+**	**Any Gr**	**Gr 1–2**	**Gr 3**	**Gr 4+**	**Any Gr**	**Gr 1–2**	**Gr 3**	**Gr 4+**
**Mucositis**	33 (97%)	31 (91%)	2 (6%)	0	13	12	1	0	20	20	0	0
**Dermatitis**	27 (79%)	27 (79%)	0	0	9	9	0	0	18	18	0	0
**Dry mouth**	8 (24%)	8 (24%)	0	0	3	3	0	0	5	5	0	0
**Dysgeusia**	6 (18%)	6 (18%)	0	0	2	2	0	0	4	4	0	0
**Tumor hemorrhage**	2 (6%)	0	2 (6%)	0	1	0	1	0	1	0	1	0
**Pneumonitis**	1 (3%)	0	1 (3%)	0	0	0	0	0	1	0	1 *	0
**Late adverse event**												
**Optic nerve disorder**	3 (9%)	3 (9%)	0	0	1	1	0	0	2	2	0	0
**Mucositis**	2 (6%)	2 (6%)	0	0	1	1	0	0	1	1 *	0	0
**Dry mouth**	2 (6%)	2 (6%)	0	0	0	0	0	0	2	2	0	0
**Dysgeusia**	2 (6%)	2 (6%)	0	0	1	1	0	0	1	1	0	0
**Trismus**	2 (6%)	2 (6%)	0	0	0	0	0	0	2	2	0	0
**Uveitis**	2 (6%)	2 (6%)	0	0	0	0	0	0	2	2 *	0	0
**Tinnitus**	2 (6%)	2 (6%)	0	0	0	0	0	0	2	2	0	0
**Dermatitis**	1 (3%)	1 (3%)	0	0	1	1	0	0	0	0	0	0
**Keratitis**	1 (3%)	1 (3%)	0	0	1	1	0	0	0	0	0	0
**Photophobia**	1 (3%)	1 (3%)	0	0	0	0	0	0	1	1	0	0
**Watering eyes**	1 (3%)	1 (3%)	0	0	0	0	0	0	1	1	0	0
**Otitis media**	1 (3%)	1 (3%)	0	0	0	0	0	0	1	1	0	0
**Oral cavity fistula**	1 (3%)	1 (3%)	0	0	0	0	0	0	1	1	0	0

**Hypothyroidism**	1 (3%)	1 (3%)	0	0	0	0	0	0	1	1 *	0	0
**Adrenal insufficiency**	1 (3%)	1 (3%)	0	0	0	0	0	0	1	1 *	0	0
**Eczema**	1 (3%)	1 (3%)	0	0	0	0	0	0	1	1 *	0	0
**Arthralgia**	1 (3%)	1 (3%)	0	0	0	0	0	0	1	1 *	0	0
**Fever**	1 (3%)	1 (3%)	0	0	0	0	0	0	1	1 *	0	0
**Tumor hemorrhage**	1 (3%)	0	1 (3%)	0	0	0	0	0	1	0	1	0
**Cataract**	1 (3%)	0	1 (3%)	0	0	0	0	0	1	0	3	0
**Trigeminal nerve disorder**	1 (3%)	0	1 (3%)	0	0	0	0	0	1	0	1	0
**Hearing impaired**	1 (3%)	0	1 (3%)	0	0	0	0	0	1	0	1	0
**Soft tissue infection**	1 (3%)	0	1 (3%)	0	0	0	0	0	1	0	1 *	0

* irAE, immune-related adverse events; ICI, immune checkpoint inhibitor; Gr, Grade
